# Addressing Healthcare Disparities Among the Homeless: Insights from a Student-Run Clinic in Houston, TX

**DOI:** 10.3390/clinpract15090161

**Published:** 2025-08-31

**Authors:** Damien Kelly, Umer Khan, Elizabeth Bixler, Gabriella Becerra, Chakema Carmack

**Affiliations:** 1 HEALTH Research Institute, Community Engagement Core, University of Houston, Houston, TX 77204, USA; 2 EnMed Program, Texas A&M College of Medicine, Houston, TX 77030, USA; 3 Texas A&M College of Medicine, Houston, TX 77030, USA; 4 Psychological, Health, & Learning Sciences Department, University of Houston, Houston, TX 77204, USA

**Keywords:** student-run clinics, healthcare access, vulnerable populations, public health interventions, unhoused individuals, social determinants of health

## Abstract

**Background:** Unhoused individuals face significant health disparities and encounter numerous barriers to accessing adequate healthcare, resulting in high rates of chronic disease, mental illness, and untreated conditions in Houston, TX. The purpose of this study was to identify prevalent health conditions within a sample of unhoused adults and to identify patterns in patient characteristics and clinical health outcomes. **Methods**: This study utilized clinical and demographic data from *n* = 191 patients who received care at a student-run clinic embedded within a homeless drop-in center in Houston, TX. Data included patient demographics, chief complaints, social determinants of health (SDOHs), past medical history, on-site diagnoses, and provider actions. **Results**: The most prevalent issues were housing insecurity (36.1%), cardiovascular conditions (38.7%), and substance use (17.8%). Nearly half of all patients (46.6%) declined treatment or left before receiving care. Significant associations were found between patient demographics and provider responses, including differences by gender and age in treatment type and diagnostic categorization. **Conclusions**: These findings underscore critical challenges in treatment adherence, diagnostic bias, and retention among unhoused populations. The study provides actionable recommendations for improving care coordination and continuity in low-barrier, student-run clinics serving medically underserved communities.

## 1. Introduction

In 2023, a total of 3270 individuals experienced homelessness in the Harris, Fort Bend, and Montgomery counties in Houston, TX, encompassing both sheltered and unsheltered populations [1]. Houston ranks second in Texas for the largest homeless population, following Dallas [2]. Homelessness is strongly correlated with poor health outcomes, as it is an independent risk factor for increased mortality, exposure to infectious diseases, and physical trauma [3,4,5]. Additionally, mental illness, which is over four times more prevalent in homeless populations, can exacerbate other chronic health conditions, such as cardiovascular disease, diabetes, obesity, asthma, and cancer [6]. Also, among unhoused youth, substance use and high-risk sexual behaviors further contribute to adverse health outcomes [7].

The COVID-19 pandemic heightened the vulnerability of unhoused individuals by exacerbating existing health risks. Limited access to healthcare and crowded living conditions led to increased rates of comorbidities, further escalating health risks during the pandemic [3,8]. Public health responses often fell short of addressing the unique needs of unhoused populations, as emergency shelters needed more capacity to provide safe and socially distanced spaces, and vaccination campaigns needed to be faster to reach these groups [9]. In the post-COVID-19 era, organizations focused on improving healthcare access for unhoused individuals are crucial in addressing these communities’ unique health challenges. Given the stark health disparities faced by unhoused populations, student-led health clinics offer a novel approach to meeting their healthcare needs. For the purposes of this study, homelessness or unhoused individuals will be defined as lacking stable housing, residing in public spaces, or living in temporary housing accommodations. Unhoused individuals encounter distinct barriers that exacerbate their medical issues and hinder their access to adequate healthcare. Several clinics have implemented personalized interventions, outreach initiatives, and technological applications, which have been shown to improve patient retention and reduce hospital visits [10].

### 1.1. Background and Previous Literature

Previous studies have identified specific barriers to care, and effective interventions aimed at improving primary care for unhoused populations highlight how addressing these challenges can reduce emergency room (ER) visits and incarceration rates while improving health outcomes. Various factors contribute to the incidence of homelessness. Job loss, drug use, intimate partner violence, mental illness, housing market conditions, and release from incarceration are all ubiquitous contributors to becoming unhoused [3,11]. One study found that conditions in the U.S. housing market have the largest influence on homelessness than any other factor [12]. The stress and living conditions of being unhoused inevitably have drastic effects on one’s health. Unsurprisingly, studies have shown that it is ideal for unhoused individuals to prioritize food and housing over social and health needs [13]. Amidst this prioritization, unhoused individuals who suffer from debilitating chronic illnesses may find it more difficult to pursue housing and/or food. Evidence shows that people without stable housing experience chronic illnesses such as asthma, chronic obstructive pulmonary disease (COPD), epilepsy, and heart conditions at rates three times higher than the general population [13]. Chronic illnesses require medical management, and unhoused individuals suffering from chronic conditions must be treated and advocated for so that they can be successful in their pursuit of better living conditions. Clinics that have attempted to alleviate the issue have found that the unhoused patient population often does not follow up and is not compliant with medications [14]. It is necessary to determine and adopt an individualized approach to treating the unhoused patient population that is easy to access and offers respect and trust [15].

Given that healthcare for unhoused individuals is marked by unique challenges and barriers, innovative interventions are necessary to improve outcomes. Federal housing policy has shifted from requiring treatment compliance before housing to adopting a “Housing First” model that allows individuals to access housing without conditions like sobriety [16]. Their work underscores the critical role of federally funded Health Care for the Homeless (HCH) projects in facilitating access to multidisciplinary care and continuity, especially for individuals reintegrating into society after incarceration or hospitalization. Expanding Medicaid has further increased insurance coverage among the unhoused, improving access to healthcare services. Recognizing the disproportionate burden of mental illness and substance use in unhoused communities, researchers recommend targeted care interventions that lead to improved satisfaction and health outcomes over standard primary care [17]. Medical respite programs and integrated care models, which combine mental health, primary care, and substance abuse treatment, are particularly effective in reducing hospitalizations and enhancing follow-up care. This highlights the importance of community partnerships and peer support in fostering positive relationships and improving care adherence.

Additionally, researchers have found the most successful primary care interventions are those that employ tailored, multidisciplinary, and collaborative care frameworks [10]. These models reduce emergency room visits, enhance chronic disease management, and improve patient satisfaction. High-performing programs often involve interdisciplinary teams, on-site social services, and community engagement, addressing both medical and social determinants of health. Similarly, there is a need to advocate for integrating medical and psychosocial services in one site, emphasizing rapport-building and logistical support to mitigate barriers such as medication nonadherence and delayed care [18].

Participatory models like the Healthcare for the Homeless Suitcase Clinic further illustrate the potential of community-based approaches [6]. By incorporating input from unhoused individuals and leveraging local resources, these initiatives address chronic and urgent care needs effectively. Mobile clinics and collaborative efforts with pharmacies and shelters reduce ER visits and enhance medication access, demonstrating the efficacy of community-driven, adaptable healthcare delivery systems. These findings underscore the importance of patient-centered, integrated care approaches in addressing the multifaceted health challenges faced by unhoused populations. Models emphasizing interdisciplinary collaboration, trust-building, and tailored interventions provide a roadmap for improving healthcare access and outcomes, particularly in community health settings like student-run clinics, such as the Texas A&M University Maroon Health Clinic, in partnership with Open Gate Homeless Ministries (Houston, TX, USA).

#### 1.1.1. Texas A&M University Maroon Health

A student-run, student-founded free clinic operated by Texas A&M Medical students has been providing care to patients at Open Gate Homeless Ministries since 2023. The clinic, open two Sundays each month year-round, offers vital healthcare services to underserved populations, particularly individuals experiencing homelessness, within a collaborative, volunteer-driven environment. Supplementary funding for the clinic’s operations was obtained from both the Texas Medical Association and the National Association of Free Clinics, which bolsters the clinic’s capacity to serve underserved populations effectively. Additional funding was secured through grants and generous donations. This collaborative support structure enables the clinic to deliver medical services while providing valuable, hands-on experience for medical students involved in community health work.

Patient health data are systematically captured through the open-source electronic medical record platform, Practice Fusion. The clinic manager, a dedicated medical student volunteer, records the number of patients served during each clinic session. Additionally, clinic attendance information is shared via the Homeless Management Information System (HMIS), facilitating broader tracking and reporting of patient demographics within the Harris County community. This data integration enhances the clinic’s capacity to contribute meaningfully to local health initiatives and address the specific needs of its unhoused patient population.

#### 1.1.2. Open Gate Homeless Ministries

Since March 2008, Open Gate has supported at-risk adults, particularly young adults, experiencing unstable housing, including those living on the streets or in shelters. Unstable housing is defined as housing that can be terminated at any time. Open Gate provides a safe, welcoming space, particularly for LGBTQ+ young adults who often face challenges in shelters or on the streets. The organization also serves young parents and their children, a demographic frequently excluded from other services due to age limits. The core principle of Open Gate is consistency and trust. For over 16 years, the organization has provided unwavering support, fostering relationships and a sense of community. Every program has emerged organically from these relationships, addressing various needs, including housing, education, mental health, and job placement. Open Gate creates a safe space for participants to express their struggles, from suicidal thoughts to grief, and offers emotional support for parents facing difficult diagnoses. The community helps individuals struggling with addiction by providing hope, acceptance, and resources.

Key services include the Sunday afternoon Hawthorne Dinners, which provide home-cooked meals alongside core services like hygiene items, clothing, diapers, bus cards, and a food pantry. The dinners also offer a vital social outlet for housed participants to maintain connections with their unhoused peers, reducing isolation and helping prevent lease violations due to overcrowding in small apartments. Additionally, Open Gate provides crucial onsite services, which are highly utilized. These include medical clinics, sexual health counseling, mental health services, HIV and substance abuse counseling, vision health, civil legal assistance, and monthly haircuts. These services are essential in addressing immediate needs, as many participants are unlikely to act on referrals to off-site providers. Through this comprehensive support, Open Gate meets the unique and varied needs of Houston’s vulnerable young adults and was an excellent infrastructure for the Texas A&M University Maroon Health Clinic.

### 1.2. Statement of the Problem and Purpose of Study

Despite various efforts, the unhoused population continues to experience higher mortality rates, increased exposure to infectious diseases, chronic health conditions exacerbated by mental illness and substance use, and persistent vulnerabilities resulting from the COVID-19 pandemic. Although some clinics have adopted innovative strategies such as personalized interventions, outreach initiatives, and other methods to improve care and patient compliance, there remains a critical need for a systematic evaluation of these approaches. This present study seeks to investigate the reach of these interventions, explore the distinct barriers unhoused individuals in Houston, TX face in accessing healthcare, and identify strategies that can bridge these gaps. The findings may provide valuable insights into primary care facilities seeking to enhance healthcare delivery and outcomes for this vulnerable population and the value of student-run clinics as a community health resource.

The objective of this study, based on the analysis of patient data from a student-run health clinic operating within an unhoused drop-in center in Houston, TX, is to identify prevalent health conditions, identify patterns in patient characteristics and clinical health outcomes, and suggest best practices for the management of student-run clinics serving homeless populations. By examining both the clinical outcomes and operational challenges, this study aims to provide an understanding of how student-led healthcare initiatives can address the unique health needs of unhoused individuals and improve care delivery in similar settings.

## 2. Materials and Methods

### 2.1. Study Site and Patients

The clinic site, Texas A&M University Maroon Health. is a student-operated healthcare clinic affiliated with Texas A&M University College of Medicine, housed within Open Gate Homeless Ministries—a nonprofit organization offering essential services to unhoused individuals, with a focus on young adults and LGBTQ+ individuals. Since 2008, Open Gate has operated as a vital community resource, providing a safe and supportive environment for Houston’s unhoused population. The clinic operates under the supervision of licensed physicians and relies heavily on volunteer medical students, offering hands-on training opportunities within a community health context. Patient care at the clinic is augmented by the on-site availability of basic health and social services provided by Open Gate, including sexual health counseling, substance use support, and civil legal assistance. This integrative approach facilitates a continuity of care while addressing both immediate medical needs and broader social determinants of health, a model increasingly recognized for improving health outcomes among unhoused individuals [3,19]. Clinic information was gathered on n = 191 patients who were unhoused clients served by Open Gate.

### 2.2. Data Collection and Measures

Clinic records from October of 2023 to March of 2025 captured patient age, gender, housing status, primary diagnoses, treatment provided, and follow-up information. Clinic providers (current medical students) also captured each patient’s chief complaints, barriers to care (e.g., social determinants of health), past medical history (PMH), differential diagnoses, and clinical actions taken during each patient’s visit. Patients were able to freely express their chief complaints and express their barriers to care (SDOH). To identify patterns across a diverse set of clinical and social variables, categorical groupings were developed based on clinical relevance, common terminology, and thematic similarities found in patient records. Researchers in the past have created similar group definitions based on symptoms and diagnosis clustering in community health and homeless care settings [17,20].

- Complaint Group: This group consisted of common complaints that participants who visited the clinic would ask the medical students to treat. The lead researcher categorized each patient’s chief complaint into seven broad diagnostic clusters (i.e., pain, respiratory, mental health, gastrointestinal, dermatological, and unclassified/multiple concerns) to align with commonly observed symptom domains in primary and urgent care settings [13,14]. Because patients were allowed to express their complaints freely, we were not able to determine the “chief” complaint amongst patients expressing multiple complaints (unless it was explicitly noted in the EMR by the medical student). Therefore, patients’ notes with multiple complaints were categorized as “multiple.” A single complaint that did not fall into the seven broad categories or complaints that were very general in nature (e.g., “not feeling well” with no other explanation) was classified as “ambiguious/other.” This allowed for the aggregation of varied symptom descriptions under unified clinical themes, improving the interpretability of complaint-based trends.
- Social Determinants of Health Group (SDOH): Unhoused and low-income participants presented with different external conditions that impact health. Medical student providers captured clinic notes regarding the context of the clinic encounter, as presented by the patients. SDOH entries were thematically organized into seven categories: housing insecurity, food insecurity, substance use, mental health/stress, disability, economic insecurity, and criminal justice history, based on well-established public health frameworks such as those outlined by the CDC and WHO. Single SDOHs were coded based on the SDOH recorded by the medical student workers. Patients who expressed multiple SDOHs, as observed in the clinic notes, were coded to indicate either 2 SDOHs or 3 + SDOHs. This structured approach highlights the intersectionality of social context with patient health needs.
- Past Medical History Group (PMH): Participants’ past medical history data were organized into eight clinically distinct categories reflecting chronic disease domains (e.g., cardiovascular, metabolic, neurological, etc.). These categories were exhaustive within the archival data. The additional categories of “multiple previous medical issues” and “none noted” were added to classify records that captured multiple past medical concerns and those with no previous medical history listed, respectively. This categorization enabled comparison across patients with similar comorbidity profiles.
- Differential Diagnosis Group: This group consists of possible causes or origins for a condition. Symptoms were grouped by systems-based categories reflecting clinical reasoning (e.g., cardiovascular, musculoskeletal, infectious, etc.). Patient records that indicated multiple diagnoses were classified as “multiple.” This supported the analysis of diagnostic patterns and appropriateness of treatment decisions.
- Action Group: Care provider actions were classified into categories representing the type of intervention (e.g., medication, counseling, referral, etc.). Medication was most frequently prescribed along with other interventions and was classified as such (e.g., “counseling + medication”). This scheme facilitated the evaluation of care delivery patterns and patient outcomes.

### 2.3. Analysis Procedure

Descriptive and inferential chi-squared tests were applied to identify significant relationships between patient demographics and health outcomes. Specifically, descriptive statistics were used to illustrate the demographic composition of the sample. For descriptive purposes, we categorized age by decade (18–29; 30–39; 40–49; 50–59; 60–69; 70–79; and 80+ years). Frequency counts and percentages were calculated to show clinic visit history, patients’ chief complaint, their associated SDOH, differential diagnoses at the time of visit, and the subsequent provider actions. This analysis aims to pinpoint the most common health issues and evaluate treatment efficacy, informing potential best practices for student-run clinics in similar settings [13,14].

To examine the association between patient-level characteristics and health outcomes, a series of chi-squared tests was conducted. We examined the independent associations between gender, ethnicity, and age with each chief complaint category, SDOH grouping, past medical history category, and past clinic visits to determine significant associations that may imply targeted efforts. Age was reported as a single value within the archival dataset. For inferential testing, we combined age groups 40–59 years as it indicates the middle-aged developmental period, and combined all age groups that were 60+ years due to the low number of individuals who were 60 years old and older. We examined the association between patient-level characteristics and provider-level outcomes. Independent chi-squared tests were conducted to test the associations between gender, ethnicity, and age with providers’ differential diagnoses and provider actions. Significant associations are delineated in the results.

### 2.4. Ethical Considerations

This study was approved by the Institutional Review Board of the University of Houston (protocol code STUDY00005174), on 4 March 2025. The application and approval included a detailed description of the retrospective study, analysis procedures, and a letter of support from the attending physician at Texas A&M University.

## 3. Results

### 3.1. Demographics and Descriptive Statistics

A total of 191 patients were included in the dataset. The mean age was 38.26 years (SD = 16.46), with a median of 31 years. The age distribution was right-skewed, with a large portion of the sample under the age of 40 years. Age categories showed that the majority of participants (38.7%) were 18–29 years old, followed by 30–39 years old (14.7%), and 50–59 years old (15.7%). Most participants identified as “Not Listed” in terms of ethnicity (60.7%), followed by Black (17.8%), Hispanic (15.2%), and White (6.3%). The gender distribution was predominantly male (61.3%), with females accounting for 34.6%, and 4.2% of participants who reported their gender as “unidentified”. Table 1 shows the demographic characteristics of the clinic patients.

Regarding clinic utilization, over half of the participants (52.4%) had never been seen previously, while 33.7% had been seen once before, and 13.9% had been seen four or more times. Regarding clinical presentations, the most frequently recorded chief complaints were pain-related issues (31.4%), followed by multiple non-singular complaints (27.2%) and ambiguous concerns (e.g., general checkup, feeling tired) (21.5%). Respiratory concerns (8.4%), gastrointestinal symptoms (7.9%), and mental health (0.5%) concerns accounted for almost 20% of primary complaints. Regarding social determinants of health (SDOHs), housing insecurity (36.1%) was the most commonly-reported determinant, followed by substance use (17.8%) and food insecurity (5.8%). Of note, 5.7% of patients spoke of two distinct social determinants, and 7.3% spoke of three or more social determinants within their clinic visit. For past medical history (PMH), cardiovascular/hypertension conditions were reported by 38.7% of patients, followed by multiple previous medical issues (15.2%), mental health/substance use (14.7%), and endocrine/metabolic disorders (8.9%). Table 2 shows the descriptive summary of the clinic visit history and chief patient complaint, and Table 3 shows the summary of patients’ reported SDOH and past medical history.

Differential diagnoses were most categorized as “multiple diagnoses” (44.6%) or unremarkable/normal (17.7%). Diagnoses related to musculoskeletal/pain accounted for 14.0% and respiratory/ENT 7.5%. The most common provider action denoted that the patient declined care or left before treatment (46.6%). Among patients who received treatment, the most frequent actions were medication provision (20.9%) and observation without treatment (11.5%). Referrals (8.9%), counseling/education (3.1%), and physical treatment, such as abscess drainage, wound dressing, etc. (3.7%), were less common. Medication co-occurred with a referral in 7.3% of the sample, and with physical treatment in 2.1% of the sample. Table 4 shows the provider-level summaries of the provider’s differential diagnoses and subsequent provider actions concluding the visit.

### 3.2. Patient-Level Associations

We examined associations between patient demographic variables and primary outcomes, including chief complaint categories, social determinants of health (SDOHs), past medical history (PMH), and frequency of clinic visits among patients attending the student-run clinic.

Complaint Group. There was a statistically significant association between complaint group and gender (χ^2^ (8) = 35.45, *p* < 0.001). This suggested that the type of presenting complaint varied significantly by gender. Specifically, of those reporting pain-related concerns, there were more male patients with this chief complaint (39 vs. 21). Of those reporting respiratory complaints, there were more males than females (11 vs. 4). This was also observed with gastrointestinal concerns, indicating more males than females reporting this concern (10 vs. 4). There were no significant associations between ethnicity and complaint group or age category and complaint group. Table 5 shows the significant associations between patient-level demographics (gender, ethnicity, and age) and health outcomes (i.e., chief complaints, SDOHs, and PMH).

Social Determinants of Health (SDOHs). When examining SDOHs, gender was significantly associated with the type of social need reported (χ^2^ (8) = 18.86, *p* = 0.016). Of those reporting a particular SDOH, substance use was more frequently reported among male patients (20 vs. 14) as well as housing insecurity (52 vs. 13), while food insecurity and SDOHs identified as “other” appeared more evenly distributed among the genders. A significant relationship was also shown between age and SDOHs when all age categories were included (χ^2^ (28) = 109.07, *p* < 0.001), indicating younger patients aged 18–29 years were more likely to report substance use (21/34 who reported substance use), while older age groups (40–59 years) had higher rates of housing insecurity (51/69 who reported housing insecurity) (Table 5).

Past Medical History (PMH). Gender was significantly associated with past medical history (χ^2^ (14) = 28.72, *p* = 0.011). Past cardiovascular conditions were more prevalent among male patients (47/73 who reported cardiovascular concerns), as was previous mental health and substance use history (22/25 who reported previous mental health issues and substance use) and previous respiratory conditions (7/8 who reported previous respiratory conditions). Females reported more past gastric or infectious disease conditions than males (7/8 who reported previous gastric and infectious disease conditions). Significant age-based differences in PMH were also identified when analyzing all age groups (χ^2^ (49) = 86.23, *p* < 0.001), with cardiovascular and endocrine conditions observed in older patients aged 40–59 years. Previous mental health and substance use conditions were more common in younger patients (*n* = 18 of 18–39 year olds vs. *n* = 10 of 40–80 year olds) (Table 5).

Clinic Utilization (Repeat Visits). Clinic utilization, measured by the number of prior visits, was significantly associated with age (χ^2^ (14) = 40.35, *p* < 0.001), with younger patients more likely to be first-time visitors and older patients more likely to have had four or more previous visits.

### 3.3. Provider-Level Associations

We examined provider-level associations between patients’ demographic variables (ethnicity, gender, age) and both clinical assessments (differential diagnoses) and provider responses (treatment actions). Table 6 shows the statistically significant associations.

Differential Diagnosis. There was a statistically significant association between differential diagnosis and gender, χ^2^ (14) = 40.94, *p* < 0.001. Males were more often diagnosed with musculoskeletal (20/26 of those receiving a musculoskeletal diagnosis) or respiratory conditions (10/14 of those receiving a respiratory diagnosis), while females more frequently received diagnoses in the GI/reproductive (6/9 of those receiving a GI or reproductive diagnosis) or neurological/mental health (4/4 of those receiving a neurological or mental health diagnosis). There was a statistically significant association between differential diagnosis and ethnicity, χ^2^ (21) = 35.39, *p* = 0.026. Notably, Black and Hispanic patients were more frequently assigned diagnoses categorized as “unremarkable/normal,” while White patients had a higher proportion of musculoskeletal/pain diagnoses.

Provider Action. Provider action (e.g., medication, referral, counseling) was significantly associated with gender, χ^2^ (10) = 21.79, *p* = 0.016. Males were more likely to receive medication (34/50 who were recommended medication), whereas females were more likely to be referred for additional services (12/17 who were referred). Age group was also significantly associated with provider action. A significant association was found between action and age category, χ^2^ (35) = 90.01, *p* < 0.001. Younger adults were more likely to receive counseling or medication, while middle-aged patients (40–59 years) were more often observed without treatment recommendations or declined care.

Provider Action by Diagnosis. There was a significant relationship between clinical action and differential diagnosis in the full model, which included all diagnostic categories, χ^2^ (35) = 122.86, *p* < 0.001. This association remained significant when excluding the “other” action group category (χ^2^ (30, *n* = 103) = 74.60, *p* < 0.001) and when excluding both “other” and “declined/left” action groups (χ^2^ (24, *n* = 59) = 68.64, *p* < 0.001). Across models, medication was most commonly provided for musculoskeletal and respiratory complaints, while observation or no treatment was most frequently documented in cases labeled as “unremarkable,” as expected.

## 4. Discussion

The present study provides descriptive results of the demographic and provider characteristics of a student-run medical clinic operating within a community drop-in facility for unhoused community members. Chief complaints and social determinants were captured, illustrating a patient population with many diagnoses and co-existing social concerns. The present study identified various significant associations between demographic characteristics (gender, age, ethnicity) and chief complaints, SDOHs, and past medical history.

The findings may be used to identify additional resources for this particular population. For instance, the findings show significantly more respiratory concerns and substance use among male individuals. This may be associated with smoking, environmental health, and/or other unidentified personal health or social issues. Having resources (such as print material or behavioral modification counselors) on hand to address environmental health or smoking cessation may be beneficial to disseminate or advise patients as they receive acute care. However, additional investigation would be needed to identify what specific messages are needed and in what format would be most helpful, given the vulnerable social conditions experienced by unhoused individuals. Additionally, student-run clinics may be optimal to address drug use. The present study found that substance use was significantly higher among both male patients and younger unhoused patients, specifically those aged 18–29 years. Early medical professional involvement is pivotal in addressing substance use concerns. Thus, student-run clinics for unhoused populations may benefit from the incorporation of drug counselors and behavioral health integration, as well as referrals for specialized treatment. Interestingly, substance use as a social determinant of health concern and a past medical history of substance abuse were both significantly observed among unhoused males in the present study. Nevertheless, this might highlight a greater societal need to address substance use concerns in unhoused populations who may have unique needs regarding substance use treatment and adherence.

The findings showed that middle-aged/older patients, aged 40–59 years, noted homelessness as a social determinant discussed during their clinic visit, and this age group was also observed to have declined treatment significantly more than the other age groups. This would be expected in an unhoused drop-in clinic; however, medical students facilitating care in such a clinic have the opportunity to interact with vulnerable populations and be exposed to patterns of health behavior that can strengthen their bedside manner and health promotion as future physicians. Insights from the present study may also lend themselves to deeper testable hypotheses. For example, based on the age group associations regarding provider actions, it could be investigated whether developing professional and kind relationships with patients, especially the most vulnerable of them (e.g., unhoused older populations), would help dispel medical mistrust and increase adherence to care in such a setting (e.g., unhoused drop-in clinics). The findings also implied that younger patients were less likely to be seen a subsequent time after their initial visit. This also emphasizes the opportunity for medical students to build patient relationships through patient retention for the care of patients throughout the lifespan.

Differences by gender and race in the diagnosis and treatment provided by the medical students operating the clinic were significant in the present study. The findings show that more Black and Hispanic patients were diagnosed as “normal” or “unremarkable,” whereas White patients were diagnosed with muscular or skeletal disorders. These findings echo previous studies on provider bias regarding pain management and diagnoses. Even when experiencing similar symptoms, meta-analytic studies have shown that Black and Hispanic patients are more likely to receive an unremarkable diagnosis [19,20], compared to White patients. Likewise, research on provider action has demonstrated implicit provider bias that favors pain medication for male patients, while female patients are less likely to be offered medication in favor of weaker pharmacological interventions when clinic presentations and symptoms are similar [21]. Studies have suggested that these differential diagnosis patterns may point to implicit provider bias rather than differences based on race and gender [19,20,21]. This may highlight an area for awareness and improvement in the future.

Notably, this study’s results highlight patterns of patients’ non-adherence to provider recommendations and a lack of adequate follow-up visits. The presence of various comorbidities along with social determinants of health highlights the importance of compliance with this patient population. This also demonstrates the necessity of advocacy by providers and medical students for a patient population that has seen low levels of medical adherence. Future studies may investigate the various phenomena that contribute to low levels of medical adherence among patients with pressing SDOHs and clinical models that may boost medical adherence among this group. Middle-aged (40–59 years) patients in the student-run clinic had a greater prevalence of housing insecurity than younger patients, which may be expected. They also demonstrated higher clinic repeat visits, which may also be expected. However, it is interesting that the same age group was reported to decline care most often. Statistically, age, as a biological factor, and homelessness, as a social determining risk factor, both worsen health outcomes. This suggests a compounding concern for medical professionals and public health. Age has been associated with medical mistrust and ethical considerations (e.g., medicine side effects, quality of life remaining) that influence older individuals’ decision to refuse treatment. Yet, the findings suggest that they are more likely than other age groups to come in for a repeat visit. This reaffirms the need for routine screening for SDOHs in clinic settings and the need for clinics to support connections in healthcare and social welfare systems [22]. Clinics serving vulnerable populations should be in close and “closed” connections with housing specialists, social workers, and behavioral health professionals, and be able to flag patients for extra support who are at high risk for severe morbidity (or mortality), for example.

Taken together, these findings may direct providers to be more cognizant of such patterns when treating different age groups and perhaps lead to better management of care. This may highlight the importance of patient-centered care, in which providers can coordinate treatment plans per behavioral patterns among different age groups with their unique SDOH and levels of medical adherence.

Medical students participating in delivering care in the free clinic had a unique opportunity to build meaningful relationships not only with their fellow students and attending physicians but also with the patients they serve. This dynamic can foster a deep sense of community and connection, bridging gaps between the healthcare system and the local population. Students were actively involved in assessing patients, ranging from long-term management of diseases to acute traumas. In this environment, they gained invaluable experience in patient care and clinical decision-making, all while working closely with attending physicians who provided guidance and mentorship. Students had the opportunity to follow some repeat patients over time, with some patients returning on a biweekly basis for ongoing care. This allowed students to observe the progression of treatment plans, adjust interventions as needed, and build long-term relationships with the individuals they are caring for. This continuity of care enhances both the educational experience for the students and the overall health outcomes for the patients, creating a mutually beneficial environment.

Medical students were able to identify pressing issues within the community and actively propose solutions to address these challenges, patient-by-patient. Through this process, they had the opportunity to gain a deeper understanding of gaps in healthcare and develop strategies to bridge those gaps among unhoused individuals and those with other vulnerable circumstances. By engaging with patients in a community setting, medical students had the opportunity to make a momentary yet tangible impact in the care of those they served. Early field experience in medicine also allows for the cultivation of essential skills for their future medical practices in diverse settings. This hands-on experience plays a crucial role in shaping them into well-rounded physicians, fostering both their clinical acumen and their ability to evaluate and address the broader social determinants of health toward community and population health.

The patterns observed in this study are consistent with findings from other student-run clinics serving medically underserved and unhoused populations. The high prevalence of cardiovascular conditions among patients aligns with data from the MSM-HEAL Clinic in Atlanta [23], where similar chronic illness patterns were observed among patients experiencing poverty and housing instability. Likewise, Broman et al. [24] documented the frequent occurrence of cardiovascular and substance use disorders across a diverse sample of student-run clinic models, suggesting a broader trend of untreated chronic illness in vulnerable populations.

The observed treatment refusal and high attrition rate—nearly half of all patients either declined care or left before treatment—mirrors national findings regarding barriers to care among unhoused populations. In previous studies, patients seen at student-run clinics expressed appreciation for staff and medical students’ empathy but also cited long wait times and disjointed coordination as reasons for disengagement. Although we did not seek to examine this notion, it is worth mentioning that our findings might reflect both logistical challenges and deeper relational issues, such as prior trauma or mistrust of institutional systems. This influences a patient’s decision to leave or avoid care. In this context, it becomes increasingly important for student-led clinics to adopt trauma-informed practices and flexible, low-barrier engagement strategies.

Differences in treatment and diagnostic coding by gender and age also suggest the possibility of implicit bias or differences in symptom presentation. Kamal et al. [25] noted that women reported lower satisfaction in student-run settings, raising questions about how demographic factors may influence provider behavior or patient perception. Though not causal, our results suggest a need for providers to be more attuned to such dynamics and to reflect critically on how age, race, gender, and SDOHs intersect with clinical decision-making.

Finally, the comparative literature strongly supports the role of interdisciplinary and integrated care models in improving outcomes for populations with complex needs. Clinics that offer on-site mental health services, social work, pharmacy access, and peer navigation show promise in reducing emergency visits and increasing continuity of care [24]. These approaches, when adapted for student-run environments, could address many of the SDOH challenges surfaced in this study. Our findings contribute to this growing body of research and highlight the unique positioning of student-run clinics to innovate care models that respond not only to medical complexity but also to structural vulnerability.

### 4.1. Implications for Practice

We suggested implications for creating a community health clinic as a part of a homeless drop-in center. For practitioners in this field, and especially for medical student development, the insights gained from this research can inform best practices for service delivery, staffing models, and patient engagement strategies tailored to the unique needs of unhoused populations.

- Healthcare practitioners and medical educators operating student-run clinics serving unhoused populations should prioritize low-barrier, relationship-based care models. The study’s findings show that nearly half of the patients either declined care or left before treatment, indicating a need for strategies focused on trust-building, continuity, and rapport. Embedding trauma-informed care principles and flexible scheduling within student-run models may improve treatment adherence and follow-up engagement.
- Public health administrators should invest in on-site, integrated services within drop-in centers and shelters. Co-locating care with other essential services, such as legal aid, hygiene, sexual health counseling, and mental health support, can address the complex social determinants of health that patients face. Partnerships with community organizations like Open Gate and mobile clinics, for example, can ensure that patients receive care at the point of need, reducing emergency room visits and improving chronic disease management.
- Medical schools should incorporate structured curricula on implicit bias, cultural humility, and social determinants of health into clinical training. This recommendation stems from the study’s findings that gender and ethnicity were significantly associated with differential diagnoses and provider actions. Practitioners-in-training should receive mentoring and reflection opportunities to prevent diagnostic disparities and promote equitable care.
- Clinics should develop targeted retention strategies for younger patients and those with substance use and mental health challenges. The data showed that younger adults presented with more behavioral health concerns, yet returned for fewer follow-up visits. Structured peer navigation, text-based appointment reminders, and community health worker outreach may improve retention in this high-risk group.
- Administrators and community partners should implement routine patient feedback systems. Regular patient satisfaction surveys or exit interviews can inform improvements in clinic operations, identify reasons for care refusal, and guide service enhancements that align with client needs. These insights are vital for student-run clinics striving to deliver responsive, patient-centered care.
- Finally, regional public health leaders and clinic partners should coordinate shared data systems to track patient outcomes over time. Integrating clinic electronic medical records (EMRs) with homeless service databases (e.g., HMIS) allows for more effective case management, identification of service gaps, and better-informed public health interventions across systems.

### 4.2. Study Limitations

Findings from the Texas A&M Maroon Health clinic may be limited due to inconclusive data from patient charts. In addition, variations in the usage of the EMR by student volunteers may exist, causing inconsistencies. Furthermore, limited data were found in the clinic’s electronic medical records (EMRs) regarding the actions the patients had taken outside the clinic to improve their health, such as consulting other facilities, utilizing resources, and taking medications as directed. The clinic is limited in its diagnostic ability, lacking the utilities to conduct services characteristic of more established primary care facilities, such as an A1c hemoglobin test, complete blood count (CBC) panel, and lipid panel. These tests would have offered valuable insight into individual patients’ progress and stages of health [26]. Additionally, it was difficult to ascertain significant associations among the ethnicities as the majority of the sample did not report their race/ethnicity. The low sample size, to date, and the limited information provided by the EMRs limited the available inferential analyses we were able to evaluate. Future research may seek to obtain a more comprehensive medical history of patients, but this may be limited by the transience of vulnerable unhoused populations. Nonetheless, the present results offer insights into the potential of student-run clinics for vulnerable populations, but are preliminary and should be taken with caution.

## 5. Conclusions

This study highlighted the complexity of healthcare delivery to unhoused populations. Disparities were observed in clinical engagement, diagnostic patterns, and provider actions within the student-run clinic setting. The findings underscore the critical need for responsive, low-barrier care models that address both clinical conditions and the broader social determinants affecting unhoused individuals. This analysis of healthcare access in Houston contributes to the growing field of community-based clinical interventions and provides actionable insights for similar socio-geographic contexts nationwide. Healthcare professionals, medical educators, and public health stakeholders may leverage these findings to advocate for policy reforms and investment in student-led and community-embedded health infrastructure. Recommendations include integrating trauma-informed care practices, expanding onsite wraparound services, and strengthening interagency data systems to enhance continuity of care. Additionally, training future providers in cultural humility and implicit bias is essential to address disparities in diagnosis and treatment.

Community-driven approaches remain vital to understanding what health services are most effective and acceptable to unhoused individuals, particularly youth and LGBTQ+ populations. Robust, multi-level strategies tailored to patient retention, engagement, and treatment adherence are urgently needed. Future research should explore the drivers of care refusal, examine long-term patient outcomes, and assess the replicability of student-run clinic models in other underserved regions. Cross-sector collaboration is essential to ensuring equitable access to healthcare for unhoused individuals and fostering sustainable change in health outcomes across vulnerable communities.

## Figures and Tables

**Table 1:** Demographic characteristics of clinic patients (*n* = 191).

Variable	Category	*n*	%
**Gender**	Male	117	61.3
	Female	66	34.6
	Unidentified	8	4.2
**Ethnicity**	White	12	6.3
	Black	34	17.8
	Hispanic	29	15.2
	Not Listed	116	60.7
**Age**	<18	7	3.7
	18–29	74	38.7
	30–39	28	14.7
	40–49	21	11.0
	50–59	30	15.7
	60–69	29	15.2
	70–79	1	0.5
	80 +	1	0.5

**Table 2:** Clinic visit history and chief complaints.

Variable	Category	*n*	%
**Times Seen** Before *	0 (never before)	98	52.4
	1–3 times	63	33.7
	4 or more times	26	13.9
**Chief** **Complaint**	Pain	60	31.4
	Respiratory	16	8.4
	Skin/Dermatological	6	3.1
	Mental Health	1	0.5
	Gastrointestinal (GI)	15	7.9
	Multiple complaints Ambiguous	52 41	27.2 21.5

* Note. Data valid for 187 cases; 4 missing.

**Table 3:** Social determinants of health and past medical history.

Variable	Category	*n*	%
**SDOH Group**	Housing Insecurity	69	36.1
	Mental Health/Stress	8	4.2
	Food Insecurity	11	5.8
	Substance Use	34	17.8
	Disability	8	4.2
	Economic Insecurity	11	5.7
	Criminal Justice History	0	0
	2 SDOHs	11	5.7
	3 + SDOHs	14	7.3
	None noted	25	13.0
**PMH Group**	Cardiovascular/Hypertension	74	38.7
	Endocrine/Metabolic	17	8.9
	Mental Health/Substance Use	28	14.7
	Respiratory	8	4.2
	Neurological/Seizure/Head Injury	2	1.0
	GI/GU/Infectious Disease	8	4.2
	Musculoskeletal/Pain	8	4.2
	Multiple medical issues	29	15.2
	None noted	17	8.9

**Table 4:** Differential diagnoses and provider actions.

Variable	Category	*n*	%
**Differential Diagnosis ^†^**	Cardiovascular/Hypertension	10	5.4
	Respiratory/ENT	14	7.5
	GI/GU/Reproductive	9	4.8
	Musculoskeletal/Pain	26	14.0
	Neurological/Mental Health/Substance Disorder	6	3.2
	Infectious/Dermatological	5	2.7
	Unremarkable/Normal	33	17.7
	Multiple	83	44.6
**Provider Action**	Counseling/Education	6	3.1
	Medication Provided	40	20.9
	Referral Only	9	4.7
	Physical Treatment Only	7	3.7
	Medication Provided + Referral	14	7.3
	Medication + Physical Treatment	4	2.1
	Observation/No Treatment	22	11.5
	Patient Declined or Left	89	46.6

Note. ^†^ Data valid for 186 cases; 5 missing.

**Table 5:** Significant chi-squared associations between patient characteristics and health outcomes.

Variable Comparison	χ^2^	Df	***p***	***n***
Complaint Group × Gender	35.45	8	<0.001 ***	191
Complaint Group × Age	29.42	35	0.734	191
Complaint Group × Ethnicity	12.43	15	0.648	191
SDOH Group × Gender	18.86	8	0.016 *	191
SDOH Group × Age	109.07	28	<0.001 ***	191
SDOH Group × Ethnicity	17.09	12	0.06	191
PMH Group × Gender	28.72	14	0.011 **	191
PMH Group × Age	86.23	49	<0.001 ***	191
PMH Group × Ethnicity	13.42	14	0.490	191
Visit Frequency × Gender	7.27	4	0.122	187
Visit Frequency × Age	40.35	14	<0.001 ***	187
Visit Frequency × Ethnicity	8.83	6	0.183	187

* significant at 0.05 alpha level; ** significant at 0.01 alpha level; *** significant at 0.001 alpha level.

**Table 6:** Significant chi-squared associations between demographic characteristics, differential diagnoses, and provider actions.

Variable Comparison	χ^2^	df	***p***	*n*
Differential Diagnosis × Gender	40.94	14	<0.001 ***	186
Differential Diagnosis × Age	61.39	49	0.110	186
Differential Diagnosis × Ethnicity	35.39	21	0.026 *	186
Provider Action × Gender	21.79	10	0.016 *	191
Provider Action × Age	90.01	35	<0.001 ***	191
Provider Action × Ethnicity	22.03	15	0.110	191
Provider Action × Diagnosis (all categories)	122.86	35	<0.001 ***	177

* significant at 0.05 alpha level; ***significant at 0.001 alpha level.

## Data Availability

The raw data supporting the conclusions of this article will be made available by the author (C.C.) on request.
